# The Blood-Brain Barrier Permeability of Lignans and Malabaricones from the Seeds of *Myristica fragrans* in the MDCK-pHaMDR Cell Monolayer Model

**DOI:** 10.3390/molecules21020134

**Published:** 2016-01-22

**Authors:** Ni Wu, Wei Xu, Gui-Yun Cao, Yan-Fang Yang, Xin-Bao Yang, Xiu-Wei Yang

**Affiliations:** State Key Laboratory of Natural and Biomimetic Drugs, Department of Natural Medicines, School of Pharmaceutical Sciences, Peking University Health Science Center, Peking University, No. 38, Xueyuan Road, Haidian District, Beijing 100191, China; 13426178575@139.com (N.W.); high-xu@163.com (W.X.); cgyxfys@163.com (G.-Y.C.); yangyanfang@bjmu.edu.cn (Y.-F.Y.); xbyang0718@163.com (X.-B.Y.)

**Keywords:** blood-brain barrier, MDCK-pHaMDR, Myristicaceae, nutmeg, *Myristica fragrans*, lignans, malabaricones

## Abstract

The blood-brain barrier (BBB) permeability of twelve lignans and three phenolic malabaricones from the seeds of *Myristica fragrans* (nutmeg) were studied with the MDCK-pHaMDR cell monolayer model. The samples were measured by high-performance liquid chromatography and the apparent permeability coefficients (*P*_app_) were calculated. Among the fifteen test compounds, benzonfuran-type, dibenzylbutane-type and arylnaphthalene-type lignans showed poor to moderate permeabilities with *P*_app_ values at 10^−8^–10^−6^ cm/s; those of 8-*O*-4′-neolignan and tetrahydrofuran-lignan were at 10^−6^–10^−5^ cm/s, meaning that their permeabilities are moderate to high; the permeabilities of malabaricones were poor as their *P*_app_ values were at 10^−8^–10^−7^ cm/s. To 5-methoxy-dehydrodiisoeugenol (**2**), *erythro*-2-(4-allyl-2,6-dimethoxyphenoxy)-1-(3,4-dimethoxyphenyl)-propan-1-ol acetate (**6**), verrucosin (**8**), and nectandrin B (**9**), an efflux way was involved and the main transporter for **6**, **8** and **9** was demonstrated to be *P*-glycoprotein. The time and concentration dependency experiments indicated the main transport mechanism for neolignans dehydrodiisoeugenol (**1**), myrislignan (**7**) and **8** was passive diffusion. This study summarized the relationship between the BBB permeability and structure parameters of the test compounds, which could be used to preliminarily predict the transport of a compound through BBB. The results provide a significant molecular basis for better understanding the potential central nervous system effects of nutmeg.

## 1. Introduction

Blood-brain barrier (BBB) is a primary interface between the central nervous system (CNS) and the systemic circulation. It is formed by the cerebral capillary endothelium and the choroid plexus epithelium and provides a highly regulated environment for the brain to function normally [1]. The properties of BBB are important considerations when designing drugs to target or avoid the brain. As a part of absorption, distribution, metabolism, excretion and toxicity (ADMET) study, drug BBB permeability evaluation can predict whether a compound or drug can get into CNS and bring therapeutic or toxic effects, which is necessary before its further development.

Several practical *in vitro* models of the brain endothelium have been developed [2], which have helped to give valuable mechanistic insights (especially into transporter function). Unlike the Madin–Darby Canine kidney (MDCK) and human colon carcinoma (Caco-2) cells [3], which are often used for predicting drug gastrointestinal absorption. No simple cell-line assay is available for BBB prediction, mainly because they are generally too leaky for permeability studies. However, the MDCK-pHaMDR cell model, established by Pastan *et al.* in 1988, is MDCK cells transfected with human multidrug resistance (MDR1) gene [4]. As it displays morphological (*i.e.*, ultrastructurally defined tight junctions), enzymatic (acetylcholinesterase, butyrylcholinesterase, gamma-glutamyl transpeptidase, superoxide dismutase, alkaline phosphatase, lactate dehydrogenase), antigenic (*i.e.*, Factor VIII) similarities with BBB as well as high expression of *P*-glycoprotein (*P*-gp), this cell model has been developed into a screening tool for drug transport in BBB [5,6,7]. Data generated on this model has shown high comparability with the results *in vivo*, while it is much easier to conduct, stable among groups, and calls for less amounts of the test sample [7,8].

Nutmeg is the seed of *Myristica fragrans* Houtt. (family: Myristicaceae). It is a well-known traditional Chinese medicine as well as a popular spice being used worldwide [9]. It was found that the extract of nutmeg showed a significant antidepressant-like effect in mice [10]. Lignans and malabaricones are two types of characteristics and the main ingredients in nutmeg and studies show that these two kinds of compounds possess various bioactivities [11,12]. For example, dehydrodiisoeugenol (**1**), a benzofuran-type neolignan, can significantly inhibit the lipopolysaccharide-stimulated activation of nuclear factor kappa B in macrophages and exhibited cytotoxicity against P-388, KB16, A549, and HT-29 cancer cell lines [13,14]. We have found that **1** and licarin B (**3**) [15], as well as an 8-*O*-4′-type neolignan, myrislignan (**7**) can also significantly inhibit nitric oxide production [16]. In addition, malabaricones B (**14**), and C (**15**) exhibit strong effects on antioxidation [17,18], anti-inflammation [19,20], antibacterium [21], anticancer [22], and inhibition of acetylcholinesterase activity [23].

Before we can further explore the possible effects inside the brain of the lignans and malabaricones from nutmeg, the BBB permeabilities of these compounds in BBB have rarely been studied. In this paper, we use the MDCK-pHaMDR cell monolayer as an *in vitro* BBB model to investigate the permeability of twelve lignans and three phenolic malabaricones isolated from nutmeg. The aim is to find out whether they can penetrate across the BBB, as well as the relationship between the structure and permeability.

## 2. Results and Discussion

The physical integrity of the MDCK-pHaMDR cell monolayer confirmed high transepithelial electrical resistance (TEER) values (1400 ± 200 Ω·cm^2^) before and after the transport. Transport ability of the qualified monolayer is in accordance with the literature as the apparent permeability coefficients (*P*_app_) values were (2.44 ± 0.11) × 10^−5^ cm/s of caffeine and (4.72 ± 0.17) × 10^−7^ cm/s of atenolol [8].

**Figure 1:**
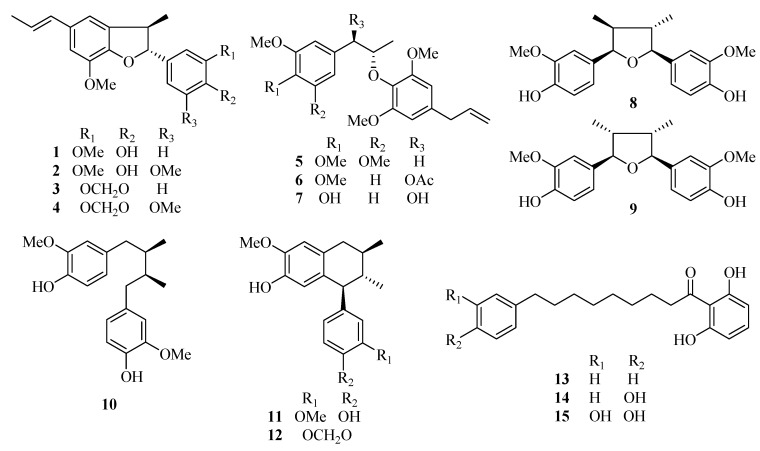
Chemical structures of the fifteen test compounds: dehydrodiisoeugenol (**1**), 5-methoxydehydrodiisoeugenol (**2**), licarin B (**3**), 3′-methoxylicarin B (**4**), *erythro*-2-(4-allyl-2,6-dimethoxyphenoxy)-1-(3,4,5-trimethoxyphenyl)-propane (**5**), *erythro*-2-(4-allyl-2,6-dimethoxyphenoxy)-1-(3,4-dimethoxyphenyl)propan-1-ol acetate (**6**), myrislignan (**7**), verrucosin (**8**), nectandrin B (**9**), dihydroguaiaretic acid (**10**), guaiacin (**11**), otobaphenol (**12**), malabaricone A (**13**), malabaricone B (**14**), and malabaricone C (**15**).

The bidirectional transport studies of malabaricones and lignans (chemical structures shown in Figure 1) were taken on this MDCK-pHaMDR cell monolayer model. The bilateral *P*_app_ values of the fifteen test compounds were summarized in Table 1.

Among these test compounds, lignans **6**–**9** exhibited high permeability with the *P*_app_ values over 10^−5^ cm/s, comparable to that of CNS-positive drug caffeine. The permeability of **1**, **2** and **5** were moderate as their *P*_app_ values are about 10^−6^ cm/s. Lignans **3**, **4**, **10**–**12** and the three malabaricones **13**–**15** showed very poor permeability, since the *P*_app_ values of **10**, **14**, **15** were at 10^−7^ cm/s, as low as that of atenolol (a CNS-negative drug), and those of **3**, **4**, **11**–**13** were even at 10^−8^ cm/s or less than their limits of quantitation.

To discuss the *P*_app_ values–structures relationship, we find that the permeabilities of lignans are related to their types of structure and substituent groups. Compounds **1**–**4**, the benzofuran-type neolignans, showed poor to moderate permeabilities; hydroxyl and methoxyl groups were more helpful to the permeability of this type of structure than methylenedioxyl group. The other two types of lignans, 8-*O*-4′-neolignan (compounds **5**–**7**) and tetrahydrofuran-type lignans (**8**,**9**), showed moderate to high permeabilities; in addition, it was easier for **7**–**9** with two hydroxyl groups to get through the MDCK-pHaMDR cell monolayer. As for dibenzylbutane-type lignan (**10**), arylnaphthalene-type lignans (**11**,**12**), and malabaricones (**13**–**15**), they can hardly get through the MDCK-pHaMDR cell monolayer, while substitution by hydroxyl group has a limited effect.

**Table 1:** The bilateral *P*_app_ values of the 15 test compounds in the MDCK-pHaMDR model and their main physicochemical properties (*n* = 3).

No.	*P*_app A→B_ (×10^−6^, cm/s)	*P*_app B→A_ (×10^−6^, cm/s)	Efflux ratio (*P*_app B→A_/*P*_app A→B_)	MW (Da)	Log D (pH 7.35)	PSA (Å^2^)	FRB	H-A	H-D
**1**	2.43 ± 0.32	1.82 ± 0.18	0.75	326.4	4.74	47.9	5	4	1
**2**	0.51 ± 0.07	1.32 ± 0.17	2.59	356.4	4.45	57.2	6	5	1
**3**	<0.062 *	<0.060 *	–	324.4	5.48	36.9	3	4	0
**4**	<0.057 *	<0.055 *	–	354.4	5.09	46.2	4	5	0
**5**	6.67 ± 0.56	8.52 ± 0.08	1.28	402.5	4.67	55.4	11	6	0
**6**	9.10 ± 0.21	21.0 ± 0.96	2.31	430.5	3.79	72.5	12	7	0
**7**	14.87 ± 0.94	13.57 ± 0.50	0.91	374.4	3.17	77.4	11	6	2
**8**	10.93 ± 0.32	20.44 ± 0.97	1.87	344.4	4.00	68.2	6	5	2
**9**	10.38 ± 1.49	14.99 ± 1.27	1.44	344.4	4.00	68.2	6	5	2
**10**	<0.145 *	<0.140 *	–	330.4	4.94	58.9	9	4	2
**11**	<0.075 *	<0.073 *	–	328.4	5.09	58.9	5	4	2
**12**	<0.073 *	<0.070 *	–	326.4	6.00	47.9	3	4	1
**13**	<0.038 *	<0.037 *	–	326.4	5.61	57.5	12	3	2
**14**	0.24 ± 0.02	0.32 ± 0.04	1.33	342.4	4.56	77.8	13	4	3
**15**	0.64 ± 0.07	0.63 ± 0.04	0.98	358.4	3.71	98.0	14	5	4

No.: Test compound number in Figure 1. *P*_app A→B_: Transport from the apical to the basolateral side. *P*_app B→A_: Transport from the basolateral to the apical side. Molecular weight (MW), polar surface area (PSA), the number of free rotatable bonds (FRB) and the number of hydrogen bond acceptors (H-A) and donors (H-D) were determined by Advanced Chemistry Development (ACD/Labs) Software V11.02. Log D values were calculated by Pallas 3.3.2.6 ADME/Tox Software. * Detected less than the limit of quantification (LOQ), so the *P*_app_ was reported as less than the value calculated according to the LOQ.

**Figure 2:**
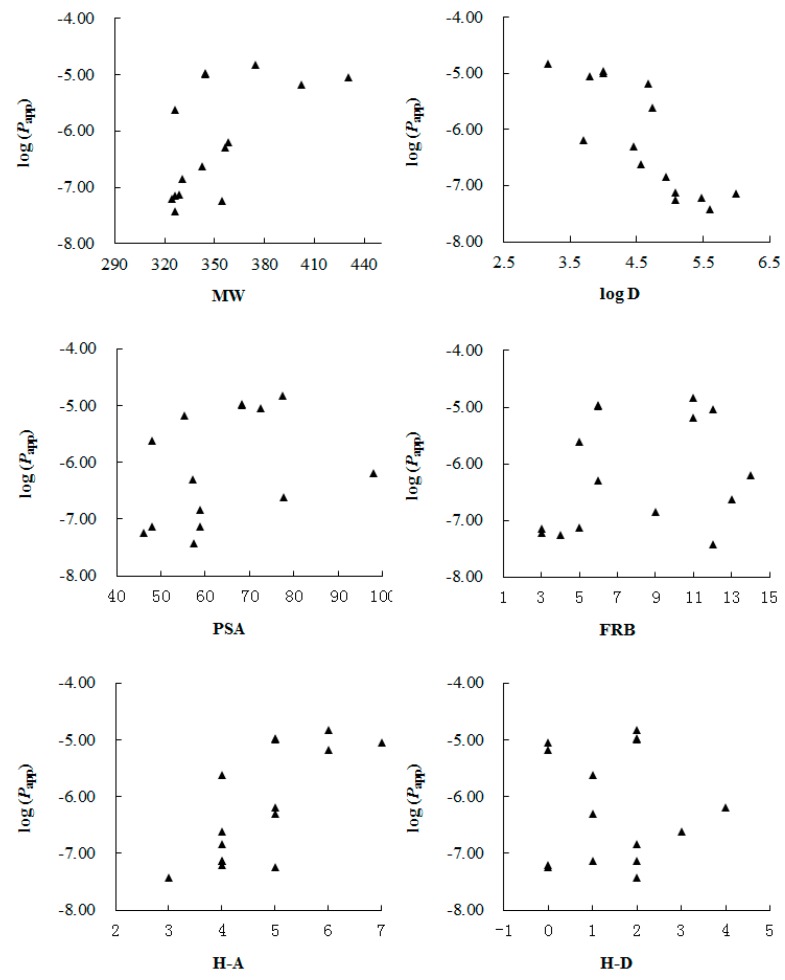
Correlation between log(*P*_app_) values of the test compouds **1**–**15** and their molecular physicochemical properties, including: molecular weight (MW), log D (at pH 7.35), polar surface area (PSA), the number of free rotatable bonds (FRB) and the number of hydrogen bond acceptors (H-A) and donors (H-D), respectively.

In addition, we correlated the *P*_app_ values with main molecular physicochemical properties including molecular weight (MW), log D (at pH 7.35), polar surface area (PSA), the number of free rotatable bonds (FRB) and the number of hydrogen bond acceptors (H-A) and donors (H-D) calculated with Origin Pro 7.5 SR1 (V 7.5776, OriginLab Corporation, Northampton, MA, USA) (Table 1). From the result shown in Figure 2, we found that log D had the hightest correlation with the *P*_app_ values, while other parameters seemed to make limited contribution to the permeability. González-Burgos *et al.* [24] have found that log D is the main determinant in the ability of diterpenes from *Sideritis* spp. across the BBB, and here we demonstrate that, for lignans and malabaricones from the seeds of *Myristica fragrans*, log D may have a great effect on the *in vitro* permeability across BBB too.

**Figure 3:**
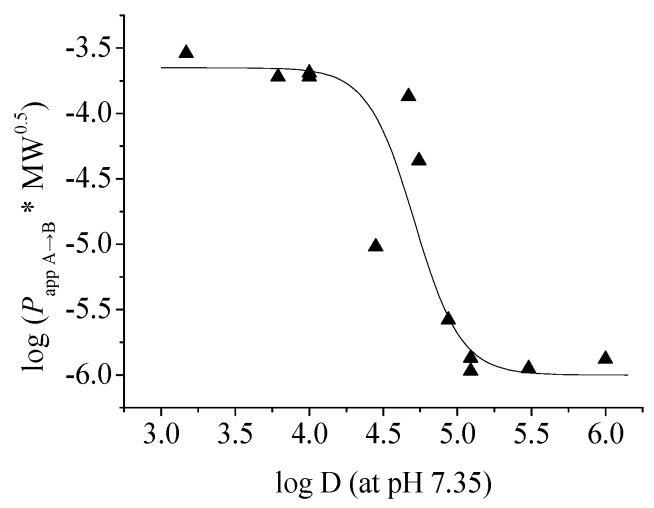
The sigmoid-like relationship between log (*P*_app A→B_ × MW^0.5^) and log D (at pH 7.35) of the twelve test lignans **1**–**12**.

To further clarify the relationship between *P*_app_ and log D, we plotted the corrected *P*_app_ values of the twelve lignans (**1**–**12**) against their log D (at pH 7.35) as described by Pardridge *et al.* [25] and got a reverse-sigmoid dependency of permeability in the log D range of 3.17–6.00 as shown in Figure 3. In the study of drug transport across the intestinal barrier with the Caco-2 cell model, Wils *et al.* [26] and our research group [27] have found that the transepithelial permeability of the compounds with moderate log D values (about 2–4) are relatively high, while those of the compounds with higher log D values (>4.5) decrease sharply and have moderate to poor permeability. The current result of the *in vitro* permeability across BBB is similar: compounds with moderate log D values (3.17–4.0) have good permeability on the MDCK-pHaMDR cell monolayer, and the permeabilities of those with high log D values (5–6) are poor. The reason for low permeability for compounds with very high log D is possibly the nonspecific binding of these compounds to the lipids of the cell monolayer.

**Figure 4:**
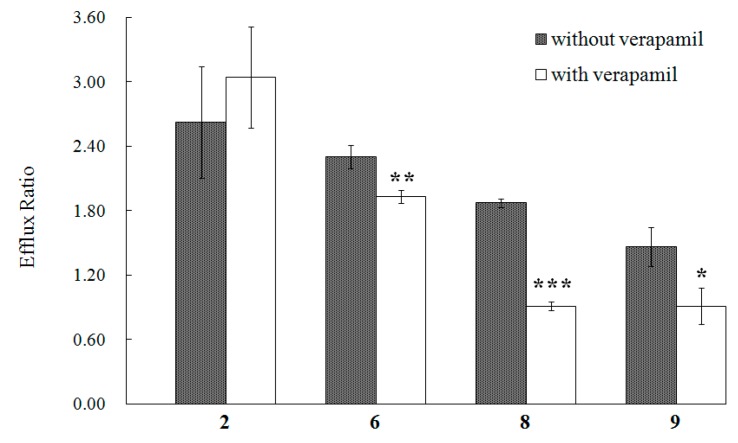
The efflux ratio of **2**, **6**, **8** and **9** in the MDCK-pHaMDR cell monolayer in the absence or presence of verapamil. Presented data are mean ± SD (*n* = 3). * *p* < 0.05, ** *p* < 0.01, and *** *p* < 0.001.

From Table 1, we can also find that the efflux ratios of compounds **2**, **6**, **8**, and **9** are much greater than 1.4, which indicates a possible efflux in the transport of these compounds [8]. As the MDCK-pHaMDR cell line was established by transfecting the human MDR1 gene into MDCK cells and selecting the cells with *P*-gp high expression, the most possible protein implementing in this efflux should be *P*-gp [4]. Thus, we conducted the transport experiments further with *P*-gp inhibitiors on these four lignans. The *P*-gp in the monolayer was demonstrated by rhodamin 123, a typical substrate of *P*-gp, as the *P*_app B→A_ value of rhodamin 123 is (3.31 ± 0.12) × 10^−6^ in the group without verapamil and (1.52 ± 0.08) × 10^−6^ with verapamil. Among the four test compounds, the efflux ratios of **6**, **8** and **9** decreased significantly in the groups with verapamil as shown in Figure 4, and the results indicated that the efflux of **6**, **8** and **9** in the MDCK-pHaMDR cell monolayer may be mainly mediated by *P*-gp. Moreover, we can infer that the efflux of **6**, **8** and **9** may happen in other cell monolayers with high *P*-gp level, such as intestinal epithelia, kidney proximal tubule epithelia and hepatocytes [28].

We also chose compounds **1**, **7** and **8**, covering the three typical types of lignans with moderate to high permeability, to further investigate their transport mechanisms by time- and concentration-dependency experiments.

**Figure 5:**
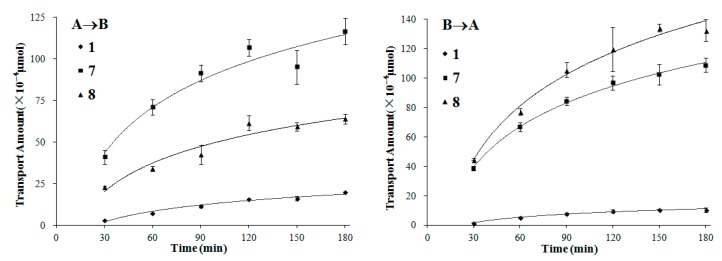
The transport amount at the different time (30–180 min). The experiment was carried out in triplicate. Data are mean ± SD. A→B: Transport from the apical to the basolateral side; B→A: Transport from the basolateral to the apical side.

In the time-dependency experiments (both A→B and B→A directions), the transport amount increased quickly during the first 90 min for all of the three lignans, and the growth of the transport slowed down gradually as time went on (Figure 5). It is in accordance with the change of the concentration gradient of the test compound between the two sides of the monolayer, as the concentration gradient was highest at first and descended along with the transport of compounds.

**Figure 6:**
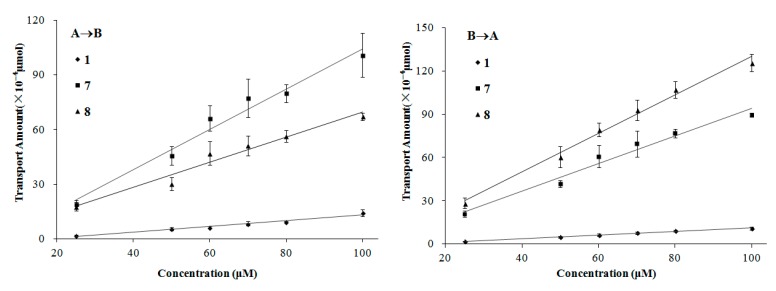
The transport amount at the different concentration (25–100 µM). The experiment was carried out in triplicate. Data are mean ± SD. A→B: Transport from the apical to the basolateral side; B→A: Transport from the basolateral to the apical side.

The transport of compounds **1**, **7**, and **8** in the MDCK-pHaMDR cell monolayer showed concentration-dependency. Meanwhile, in the concentration-dependency experiments, the transport amount increased nearly linearly on the bidirectional permeations for the three lignans (Figure 6). It is also a symbol of passive diffusion with the character of concentration-dependency [29]. According to these results, we suggest that the transport mechanism of these three kinds of lignans is mostly passive diffusion. It should also be taken into consideration that, to some special molecules, such as compound **8**, the efflux mechanism by *P*-gp will be partly involved as mentioned above.

## 3. Experimental Section

### 3.1. Materials and Reagents

Dehydrodiisoeugenol (**1**), 5-methoxydehydrodiisoeugenol (**2**), licarin B (**3**), 3′-methoxylicarin B (**4**), *erythro*-2-(4-allyl-2,6-dimethoxyphenoxy)-1-(3,4,5-trimethoxyphenyl)propane (**5**), *erythro*-2-(4-allyl-2,6-dimethoxyphenoxy)-1-(3,4-dimethoxyphenyl)propan-1-ol acetate (**6**), myrislignan (**7**), verrucosin (**8**), nectandrin B (**9**), dihydroguaiaretic acid (**10**), guaiacin (**11**), otobaphenol (**12**), malabaricone A (**13**), malabaricone B (**14**) and malabaricone C (**15**) were isolated from nutmeg by our research group (unpublished work) and were identified on the basis of spectral data [12,30,31]. The chemical structures of the 15 compounds are shown in Figure 1. Their purities were above 98% measured by reversed-phase high performance liquid chromatography (RP-HPLC).

Dulbecco's modified Eagle's medium (DMEM), fetal bovine serum (FBS), 0.25% trypsin-EDTA, phosphate buffered saline (PBS), other culture media and supplements were purchased from Gibco (Life Science Technologies, Carlsbad, CA, USA). Dimetheylsulphoxide (DMSO), atenolol, rhodamine 123, (±)-verapamil hydrochloride, and 3-(4,5-dimethyl-2-thiazolyl)-2,5-diphenyl-2*H*-tetrazolium bromide (MTT) were obtained from Sigma-Aldrich (St. Louis, MO, USA). Colchicine was from Sinopharm Chemical Reagent Co., Ltd. (Beijing, China). Caffeine was from National Institute of Metrology (Beijing, China). Reagents for Hank's Balanced Salts Solution (HBSS) and other chemicals for analytical grade were purchased from Beijing Chemical Works (Beijing, China). The solvents used in HPLC were of HPLC grade (J. T. Backer, Center Valley, PA, USA). Milli-Q water (Millipore, Bedford, MA, USA) was used throughout the study. Cell culture flasks and Transwell^®^ plates were purchased from Corning Inc. (Cambridge, MA, USA).

### 3.2. Cell Culture

The MDCK-pHaMDR cell line was a gift from Dr. Michael M. Gottesman (National Institute of Health, Bethesda, MD, USA). The cytotoxicity of the test compounds on the MDCK-pHaMDR cells was determined by the MTT assay on a Thermo Multiskan MK3 Automated Microplate Reader (Thermo-Labsystems, Franklin, MA, USA). This cell line was cultured at 37 °C in 5% CO_2_ using DMEM with d-glucose (4.5 g/L), NaHCO_3_ (3.7 g/L) and sodium pyruvate (110 mg/L), supplemented with 10% FBS and 80 ng/mL of colchicine. The passage numbers of the cells in this study were between 12 and 20.

### 3.3. Transport Studies and Sample Preparation

The MDCK-pHaMDR cells were seeded at a density of about 8 × 10^4^ cells/mL on the 12-well Transwell^®^ plates. After an eight-day culture, the cells reached confluence and differentiation for the transport study.

HBSS (pH 7.35) was used as the transport medium. On the 8th day, the monolayers were washed twice and shaken at 55 rpm for 30 min with HBSS at 37 °C. Then, the TEER was measured with an epithelial volt-ohm meter (EVOM; World Precision Instrument) to examine the monolayer's integrity. Only those with the TEER values above 1000 Ω·cm^2^ were qualified for the transport.

The stock solutions of the fifteen test compounds, caffeine, rhodamine 123 and (±)-verapamil hydrochloride were prepared at 10 mM and atenolol at 20 mM in DMSO. The stock solution was further diluted to designed concentration by HBSS. According to the results in preliminary experiments, the highest concentration of DMSO was set as 1%.

The bidirectional transport assay was initiated by adding a test compound to either the apical (0.5 mL for apical to basolateral transport, A→B) or the basolateral (1.5 mL for basolateral to apical transport, B→A) side. After shaking at 55 rpm and 37 °C for a certain time, samples (A→B: 400 µL from the apical side and 1300 µL from the basolateral side; B→A: 450 µL from the apical side and 450 µL from the basolateral side) were collected, immediately frozen, lyophilized and preserved below –20 °C before analysis. TEER values were measured again to demonstrate the monolayer's integrity at the end of the experiment.

In the single-time and -concentration experiments, the concentration was 100 µM for the fifteen test compounds and the incubation time was chosen as 90 min according to both the dissolubility of the compounds in HBSS and the detection sensitivity of analytical methods. Compounds **1**, **7** and **8**, representing three typical types of lignans, were further chosen to investigate the transport mechanisms by observing their time- and concentration-dependency. In the time-dependency experiment, test compounds at 100 µM were added in the donor side and was sampled from 30 to 180 min. In the concentration-dependency experiment, the concentration range was set from 25 to 100 µM and the time was 90 min. The control experiments on CNS-positive drug caffeine and CNS-negative drug atenolol were also conducted to examine the integrity and transportation ability of the monolayer [8].

As for compounds **2**, **6**, **8** and **9**, whose efflux ratios were more than 1.4, transport experiments with *P*-gp inhibition were conducted to define the underlying mechanisms. Verapamil at 100 µM as a selective inhibitor of *P*-gp was added into HBSS [32,33]. The cells were pre-incubated with the inhibitor for 30 min and then the test compounds at 100 µM were loaded as described above. Rhodamine 123, a typical substrate of *P*-gp, was chosen as the control group.

### 3.4. HPLC Analysis

An Agilent 1260 series HPLC system (Agilent Technologies, Palo Alto, CA, USA) consisting of a G1311C pump, a G1329B injector, a G1316H TCC, a G1315D DAD detector and an Agilent ChemStation (Version B.04.03) was used. An analytical Diamonsil^®^ ODS C_18_ column (250 × 4.6 mm i.d., 5 µm) with a C_18_ guard cartridge (8 × 4.6 mm i.d.) (Dikma Technology, Inc., Beijing, China) was employed. The flow rate was 1.0 mL/min and the system temperature was 25 °C. HPLC methods for the test compounds were shown in the Supplementary Materials section (Tables S1 and S2) and the methodological evaluation showed good precision and accuracy.

As for quantification, the lyophilized samples were dissolved in methanol of proper volume, thoroughly vortex-mixed, ultrasonicated at 20 KHz for 20 min and then centrifuged at 16,000× *g* for 10 min. An aliquot of 50 µL supernatant was used for the assay. The lyophilized cell monolayers were ultrasonicated with 200 µL 70% methanol and then treated as above.

### 3.5. Data Analysis

*P*_app_ of each compound was calculated based on the following equation:

(1) *P*_app_ = (ΔQ/Δt)/(AC_0_) (cm/s)

where ΔQ/Δt is the appearance rate of the test compound at the receiver side (µmol/s), C_0_ is the initial concentration of the test compound at the donor side (µM), and A is the membrane surface area (cm^2^). All of the experiments were conducted in triplicate.

### 3.6. Statistical Analysis

The data are presented as mean ± standard deviation (SD). Statistical differences were assessed by Student's *t*-test with Excel 2003 (Microsoft Corp., Seattle, WA, USA). The *p* values less than 0.05 were regarded as statistically significant.

## 4. Conclusions

The MDCK-pHaMDR cell monolayer model has been used for predicting BBB permeability of small molecular compounds or drugs [34,35]. This study on the bidirectional transport of the main fifteen compounds isolated from the seeds of *Myristica fragrans* in the MDCK-pHaMDR cell monolayer model showed that the *P*_app_ values of benzonfuran-type, dibenzylbutane-type and arylnaphthalene-type lignans were at 10^−8^–10^−6^ cm/s that indicated poor to moderate permeabilities in BBB; those of 8-*O*-4′-neolignan and tetrahydrofuran-lignan were at 10^−6^–10^−5^ cm/s, meaning their permeabilities were moderate to high; malabaricones showed poor permeability with *P*_app_ values at 10^−8^–10^−7^ cm/s. An efflux way was involved in the transport of compounds **2**, **6**, **8**, and **9**, among which *P*-gp was the main transporter for the efflux of **6**, **8**, and **9**. The primary transport mechanism for lignans **1**, **7** and **8** was passive diffusion. These results help us summarize the relationship between the permeability and structure, by which the drug transport through BBB can be preliminarily predicted. The results provide a significant molecular basis for better understanding the potential CNS effects of nutmeg. However, whether these compounds can actually enter the brain and play a role in the CNS requires further *in vivo* study.

## Supplementary Materials

HPLC methods for the test compounds are available as Supplementary Materials, which may be accessed at: http://www.mdpi.com/1420-3049/21/2/134/s1.
