# Supplementary material for: The Blood-Brain Barrier Permeability of Lignans and Malabaricones from the Seeds of Myristica fragrans in the MDCK-pHaMDR Cell Monolayer Model
Source: Molecules. 2016 Jan 22;21(2):134. doi: 10.3390/molecules21020134 (PMC6274353; doi:10.3390/molecules21020134)
Supplement: Supplementary file 1 [file molecules-21-00134-s001.pdf]

Supplementary Materials: The Blood-Brain Barrier Permeability of Lignans and Malabaricones from the Seeds of *Myristica fragrans* in the MDCK-pHaMDR Cell Monolayer Model

Ni Wu, Wei Xu, Gui-Yun Cao, Yan-Fang Yang, Xin-Bao Yang and Xiu-Wei Yang *

Table S1. The mobile phase, retention time, detection wavelength of HPLC method and linear equation, correlation coefficient (r^2), linear range, LOQ and LOD for the test compounds.

Compound	Mobile Phase (*v*/*v*)	Retention Time (min)	Detection Wavelength (nm)	Linear Equation (*y* Peak Area; *x* Concentration, μM)	r^2	Linear Range (μM)	LOQ (μM)	LOD (μM)
**1**	MeOH–H_2O 78:22	12.3	270	$y = 0.0195x - 0.1096$	0.9994	0.25–200.00	0.049	0.015
**2**	MeOH–H_2O 90:10	4.8	274	$y = 0.0468x - 1.5821$	0.9992	0.25–200.00	0.058	0.018
**3**	MeOH–H_2O 90:10	7.9	274	$y = 0.0258x - 1.8230$	0.9990	0.125–200.00	0.122	0.037
**4**	MeOH–H_2O 90:10	7.3	274	$y = 0.0332x - 2.4962$	0.9990	0.125–200.00	0.121	0.036
**5**	MeOH–H_2O 83:17	7.4	274	$y = 0.2535x - 2.3293$	0.9991	5.00–200.00	4.429	1.329
**6**	MeOH–H_2O 90:10	4.2	274	$y = 0.1940x - 2.4585$	0.9990	0.25–200.00	0.040	0.012
**7**	MeOH–H_2O 77:23	5.9	272	$y = 0.0998x + 0.1368$	0.9996	0.50–200.00	0.450	0.135
**8**	MeOH–H_2O 87:13	3.6	274	$y = 0.0922x - 0.4613$	0.9993	0.25–200.00	0.025	0.008
**9**	MeOH–H_2O 87:13	3.7	274	$y = 0.0942x - 0.3602$	0.9996	0.25–200.00	0.019	0.006
**10**	MeOH–H_2O 85:15	5.7	274	$y = 0.4497x - 1.6266$	0.9993	0.50–200.00	0.484	0.145
**11**	MeOH–H_2O 85:15	6.1	274	$y = 0.0787x - 0.3489$	0.9991	0.25–200.00	0.254	0.076
**12**	MeOH–H_2O 85:15	8.2	274	$y = 0.0904x + 1.3975$	0.9992	0.25–200.00	0.241	0.072
**13**	MeOH–H_2O (with 0.4% acetic acid) 89:11	12.6	272	$y = 0.0374x - 0.3967$	0.9991	0.125–200.00	0.125	0.038
**14**	MeOH–H_2O (with 0.4% acetic acid) 83:17	9.2	272	$y = 0.0324x - 0.0880$	0.9995	0.125–200.00	0.124	0.037
**15**	MeOH–H_2O (with 0.4% acetic acid) 85:15	5.7	272	$y = 0.0393x + 0.0085$	0.9994	0.125–200.00	0.089	0.027
caffeine	MeOH-H_2O 40:60	5.0	272	$y = 0.0551x + 0.0154$	0.9995	5.00-25.00	4.743	1.423
atenolol	CH_3CN–H_2O (with 0.2% phosphoric acid) 8:92	6.2	204	$y = 0.0723x + 2.8512$	0.9993	4.00-200.00	3.996	1.199
rhodamine 123	MeOH–H_2O 40:60	4.8	210	$y = 0.0017x + 0.0018$	0.9993	0.20-10.00	0.183	0.055

Table S2. The precision, accuracy and recovery for the test compounds (*n* = 3)

Compound	Concentration (μM)	Precision (RSD, %)		Accuracy (%)		Recovery (%)	
		Intra-day	Inter-day	Intra-day	Inter-day	Mean	RSD
**1**	10	2.17	3.74	98.51	100.37	95.36	2.43
	50	2.64	2.85	95.26	96.75	93.95	3.37
	100	1.58	3.02	100.49	99.58	98.84	2.81
**2**	10	3.36	4.73	97.39	99.26	97.59	1.79
	50	2.11	4.11	102.56	105.68	99.86	0.92
	100	2.04	3.98	103.61	101.96	98.50	2.63
**3**	10	1.96	2.74	99.41	101.63	101.55	2.97
	50	3.41	3.92	97.58	98.21	99.61	3.23
	100	2.88	2.98	98.82	101.38	97.95	1.59
**4**	10	2.61	3.49	95.63	99.05	100.35	2.07
	50	1.40	4.07	100.74	103.86	104.38	4.21
	100	1.37	3.28	99.58	96.45	96.59	3.63
**5**	10	3.53	5.01	96.79	99.30	103.25	2.40
	50	2.95	3.37	97.38	96.49	99.57	2.93
	100	3.73	3.62	95.41	97.73	100.28	1.67
**6**	10	4.49	4.71	103.43	105.29	101.96	2.95
	50	1.02	2.39	99.73	97.48	98.26	3.26
	100	3.14	3.53	98.40	102.35	99.58	2.74
**7**	10	3.68	3.94	96.77	97.93	96.05	2.84
	50	2.37	2.69	98.59	95.47	96.37	1.79
	100	1.72	3.08	98.71	100.83	99.25	2.41
**8**	10	2.39	4.21	102.37	100.59	104.81	3.04
	50	2.31	3.49	101.79	103.33	102.47	2.53
	100	1.88	3.00	99.85	102.87	101.26	2.66
**9**	10	3.49	2.99	97.37	99.21	98.53	2.75
	50	4.03	3.58	98.52	100.03	100.39	1.93
	100	3.84	3.75	97.73	98.39	99.02	4.22

Table S2. *Cont.*

Compound	Concentration (μM)	Precision (RSD, %)		Accuracy (%)		Recovery (%)	
		Intra-day	Inter-day	Intra-day	Inter-day	Mean	RSD
**10**	10	4.56	3.22	98.59	100.54	99.13	2.09
	50	1.99	2.00	96.49	98.17	101.68	2.85
	100	2.06	2.48	102.97	103.27	99.89	1.56
**11**	10	5.01	4.79	94.28	94.97	95.85	2.48
	50	3.37	3.44	96.71	99.93	96.95	3.02
	100	2.15	4.20	95.49	97.36	98.01	3.87
**12**	10	3.23	3.01	96.37	97.29	101.76	3.69
	50	1.62	1.76	98.66	100.07	99.74	2.16
	100	2.08	2.27	96.11	96.04	98.92	0.92
**13**	10	3.67	4.57	99.20	103.44	100.73	0.99
	50	2.18	3.98	103.64	100.39	103.21	2.53
	100	3.02	3.38	102.73	103.56	105.26	1.68
**14**	10	4.28	5.24	96.32	98.49	100.03	2.42
	50	3.44	3.81	99.68	95.35	99.46	3.33
	100	3.26	4.49	97.13	99.30	95.96	2.05
**15**	10	3.29	3.22	98.83	103.15	101.39	2.73
	50	1.03	3.07	104.72	100.35	100.29	1.79
	100	3.16	4.10	99.43	102.42	96.98	2.06
caffeine	5	1.76	2.63	99.37	102.46	101.43	3.36
	10	2.48	4.01	100.64	99.76	98.26	2.94
	20	2.51	3.15	98.58	101.23	97.30	2.35
atenolol	10	3.17	3.02	96.65	97.53	95.68	2.77
	50	2.46	4.79	97.29	99.41	99.87	1.82
	100	2.98	3.75	96.82	97.35	96.24	2.20
rhodamine 123	1	4.23	4.73	97.25	105.00	98.75	2.39
	5	2.59	1.87	100.30	101.80	101.10	2.71
	10	1.08	1.79	98.13	101.15	99.88	1.77
